# Malignant transformation and genetic alterations are uncoupled in early colorectal cancer progression

**DOI:** 10.1186/s12915-020-00844-x

**Published:** 2020-09-07

**Authors:** Soulafa Mamlouk, Tincy Simon, Laura Tomás, David C. Wedge, Alexander Arnold, Andrea Menne, David Horst, David Capper, Markus Morkel, David Posada, Christine Sers, Hendrik Bläker

**Affiliations:** 1 Institute of Pathology, Charité Universitätsmedizin Berlin, Berlin, Germany; 2 German Cancer Consortium (DKTK), Heidelberg, Germany; 3 BSIO Berlin School of Integrative Oncology, University Medicine Charité, Berlin, Germany; 4 Department of Biochemistry, Genetics, and Immunology, University of Vigo, Vigo, Spain; 5 Biomedical Research Center (CINBIO), University of Vigo, Vigo, Spain; 6 Galicia Sur Health Research Institute, Vigo, Spain; 7 Big Data Institute, University of Oxford, Oxford, UK; 8 Oxford NIHR Biomedical Research Centre, Oxford, Germany; 9 Manchester Cancer Research Centre, University of Manchester, Manchester, UK; 10 Institute of Neuropathology, Charité Universitätsmedizin Berlin, Berlin, Germany; 11 Department für Diagnostik, Institut für Pathologie, Universitätsklinikum Leipzig AöR, Leipzig, Germany

**Keywords:** Adenoma, Carcinoma, Cancer progression, Cancer driver mutations, TP53 mutations, Copy number alterations, Polyp

## Abstract

**Background:**

Colorectal cancer (CRC) development is generally accepted as a sequential process, with genetic mutations determining phenotypic tumor progression. However, matching genetic profiles with histological transition requires the analyses of temporal samples from the same patient at key stages of progression.

**Results:**

Here, we compared the genetic profiles of 34 early carcinomas with their respective adenomatous precursors to assess timing and heterogeneity of driver alterations accompanying the switch from benign adenoma to malignant carcinoma. In almost half of the cases, driver mutations specific to the carcinoma stage were not observed. In samples where carcinoma-specific alterations were present, *TP53* mutations and chromosome 20 copy gains commonly accompanied the switch from adenomatous tissue to carcinoma. Remarkably, 40% and 50% of high-grade adenomas shared *TP53* mutations and chromosome 20 gains, respectively, with their matched carcinomas. In addition, multi-regional analyses revealed greater heterogeneity of driver mutations in adenomas compared to their matched carcinomas.

**Conclusion:**

Genetic alterations in *TP53* and chromosome 20 occur at the earliest histological stage in colorectal carcinomas (pTis and pT1). However, high-grade adenomas can share these alterations despite their histological distinction. Based on the well-defined sequence of CRC development, we suggest that the timing of genetic changes during neoplastic progression is frequently uncoupled from histological progression.

## Background

It is estimated that the global burden of colorectal cancer (CRC) will reach 2.2 million new cases by 2030, leading to more than one million cancer-related deaths [1]. Most CRCs follow a well-defined adenoma-to-carcinoma sequence, beginning as a polyp and progressing to invasive metastatic disease. Adenomas are classified as low- or high-grade intraepithelial neoplasia depending on the extent and severity of their histological changes. Some adenomas undergo spontaneous regression, while others progress to carcinoma [2]. The earliest and potentially life-threatening stage of carcinoma is pT1, characterized by the presence of cancer glands that have invaded the submucosal layer of the bowel wall. These cancers are preceded by high-grade, intra-mucosal neoplasia, which may still grow in an adenomatous fashion or may already share typical cytological and/or architectural features of malignancy, including mucosal invasion. The latter are considered unable to metastasize, as indicated by their categorization as high-grade intraepithelial neoplasia by the WHO and as carcinoma in situ (pTis) by the UICC. Both pT1 and pTis carcinomas are occasionally found in endoscopically resected polyps together with remnants of their surrounding benign precursors.

Based on genome integrity and mutation load, CRC tumors can be classified as either microsatellite stable (MSS) or unstable (MSI). MSS CRCs typically harbor mutations in *APC*, *KRAS*, and *TP53* [3–6], and copy number alterations (CNAs) in chromosomes 7, 8, 18, and 20 [7]. In contrast, MSI tumors display fewer CNAs and, particularly when sporadic, a much higher somatic mutational load. Typical mutations include *BRAF*, an alternative initiating mutation in the MSI progression route involving serrated adenoma, and TGFBR2 [8, 9].

Long-standing evidence supports CRC development as a stepwise progression that involves the accumulation of CRC driver mutations [10, 11]. The Vogelstein and Fearon MSS model identifies the loss of *APC/β-catenin* in the initial formation of adenoma polyps, mutations in *KRAS* in the developing adenoma, and *TP53*, *FBXW7*, *TCF7L2*, and *PTEN* together with the loss of chromosome 18q in carcinomas. *TP53* mutations have long been considered facilitators of the transition to malignancy in CRC and are closely associated with the adenoma-carcinoma transition [11, 12]. Although CRC driver alterations are well-investigated in the late-stage disease, a key challenge remains in identifying the order of their appearance during the early stages of tumorigenesis.

Here, we sought to follow the transition of individual adenomas into carcinomas and monitor the driver alterations involved at the early stages of development. For this, we used patient polyp and colonic segment resections with adenomas that were captured transforming into carcinoma. These tumor-matched samples additionally provided us with a valuable tool to investigate clonal evolution and selection [13] at different stages of progression [14], accompanied by histological evidence of oncogenic transformation. Our work combines multi-regional adenoma-carcinoma samples, high-depth panel sequencing, and evolutionary analyses to provide a detailed view of the branching point between benign and malignant colonic neoplasms.

## Results

**Fig. 1:**
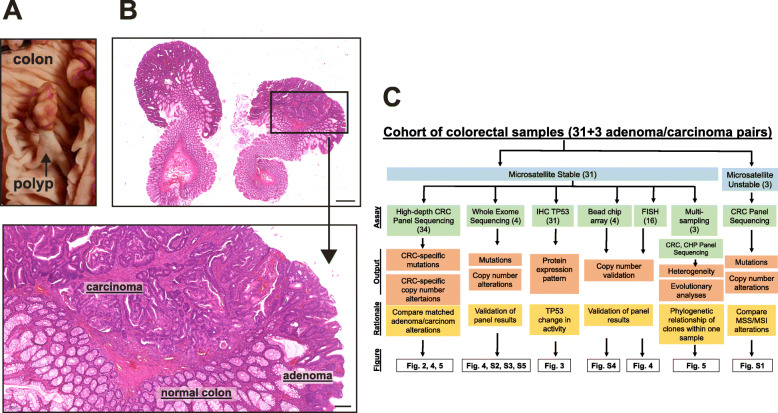
Matched adenoma-carcinoma samples. **a** Macroscopic view of a pedunculated polyp in the colon (sample AC20). **b** Microscopic view of a bisected polypectomy specimen containing both adenoma and carcinoma components seen at low magnification. Hematoxylin and eosin (H&E) staining. Scale bar 1000 μm. The arrow points to a higher magnification (× 5) of the area marked where normal colon, remnant adenoma, and early carcinoma components are visible. Scale bar 200 μm. For DNA analyses, adenomatous and carcinomatous components were separately scratched from the same sample and sequenced with a colorectal cancer (CRC)-specific panel. **c** Experimental layout of our study, indicating assays used, the output we can expect, and why we chose each assay. The numbers of samples employed for each assay are in brackets. IHC, Immunohistochemistry; FISH, fluorescent in situ hybridization; CHP, cancer hot spot

To compare early carcinomas with their adenomatous precursors, we isolated matched adenomatous and carcinomatous tissue from the same sample (Fig. 1a, b; Additional file 1: Figure S1A). All samples were formalin-fixed and paraffin-embedded (FFPE). A quality cutoff of *Q* = 50 and allele frequency cutoff of 8% were used to ensure FFPE artifacts were eliminated (see the “Methods” section and Mamlouk et al. [7] for details). Cohort details can be found in Table 1 and in Additional file 2. An overview of the different assays employed in this study is displayed in Fig. 1c.

**Table 1:** Cohort of adenoma-carcinoma pairs from 34 samples investigated

Cohort				Microsatellite stable (MSS) lesions			
CRC		Excision method		Adenoma grade		Carcinoma stage	
MSS	31	Polypectomy	22	Low grade	16	pTis	11
MSI	3	Resection	12	High grade	15	pT1	18
						pT2	2

MSS samples were further grouped as primary tumor in situ (pTis), primary tumor stage 1 (pT1), or primary tumor stage 2 (pT2)

*MSS* microsatellite stable, *MSI* microsatellite instable

### Driver mutations can be shared by adenoma-carcinoma pairs

**Fig. 2:**
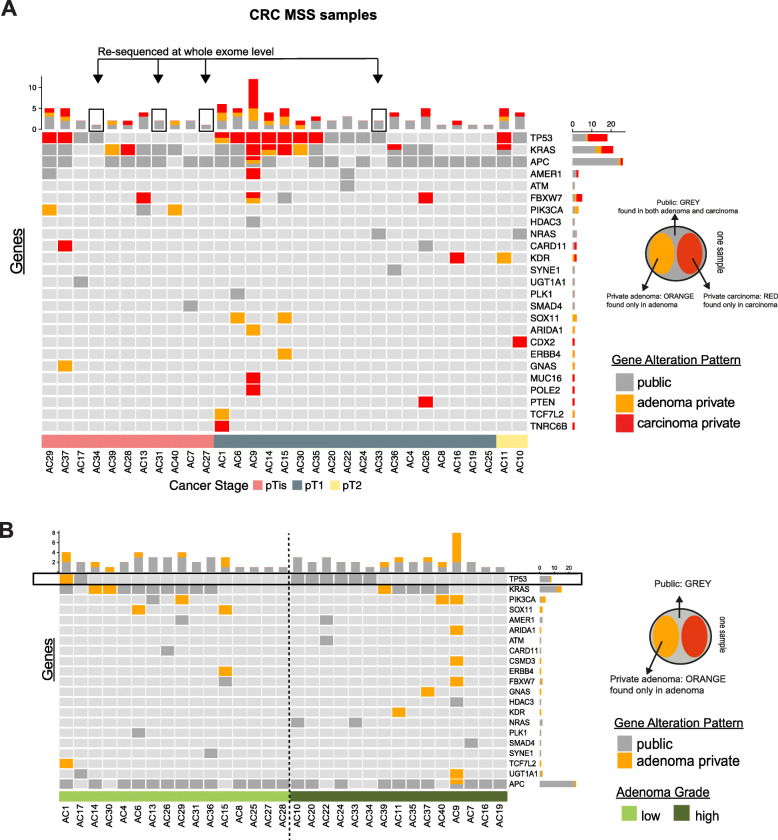
Driver mutations are both private and shared between matched adenoma-carcinoma samples. **a** High-depth panel sequencing results of 100 most commonly altered CRC genes. Mutations in samples are public (gray) if the identical mutation is found in both the adenoma and the carcinoma belonging to one sample (matched samples). Mutations found exclusively in the adenoma or the carcinoma are “private” and depicted in orange or red, respectively (see insert on the right for color depiction). Each column represents one sample with matched adenoma and carcinoma tissue. Four samples which contained no private mutations were further re-sequenced by whole-exome sequencing (WES; arrows). Samples are grouped according to cancer stage at the time of isolation. **b** Samples grouped according to adenoma grade (low- and high-grade dysplasia) from each sample, only alterations found in the adenoma are displayed (that is, public and adenoma-private mutations, in gray and orange, respectively) (see insert bottom right for color depiction). Note: only microsatellite stable (MSS) samples are displayed. Only non-synonymous mutations are displayed

To identify driver mutations responsible for CRC progression, we performed high-depth sequencing using a CRC-specific panel [7] on 31 MSS and 3 MSI, and whole-exome sequencing (WES) on 4 MSS samples. *APC* was the most commonly mutated gene, identified in 77% of the MSS cases, in line with its known role as the adenoma-initiating mutation in CRC (Fig. 2a). Two of the three MSI samples, but none of the 31 MSS samples, contained an activating *BRAF* mutation (Additional file 1: Figure S1B). The *BRAF*-negative serrated adenoma (AC43) displayed a truncating *ATM* mutation shared with the matched carcinoma. Initiating *APC* and *BRAF* mutations were usually *public* (shared between matched adenoma and carcinoma). Sample AC9 displayed a hyper-mutated profile with several adenoma- and carcinoma-specific *APC* missense mutations, along with a public non-sense *APC* mutation. Further results will focus solely on MSS tumors unless otherwise stated.

To identify genetic alterations responsible for the switch from adenoma to early-stage carcinoma, we compared the mutational burden of adenomas and their matched carcinomas using a 100-gene CRC-specific panel. We found that *TP53* was mutated most often in the carcinoma and not in the matched adenoma (32% cases) (termed *carcinoma-private* henceforth), followed by *KRAS* (19%) and *FBXW7* (9.6%). However, these three genes also harbored some *adenoma-private* mutations; *KRAS* was mutated in the adenomatous regions of AC14 (p.G12D), AC30 (p.G12D), and AC39 (p.G12A) but not in their matched carcinoma. In AC14, a different *KRAS* mutation was *carcinoma-private* (p.G12C) even though these adenoma and carcinoma tissues shared a common ancestor, as suggested by a public mutation in *APC* (p.R1114*) (mutation list in Additional file 3). *FBXW7* was mutated in four samples and was *carcinoma-private* in three of these. The hypermutated AC9 sample had both *adenoma-private* (p.S582L) and *carcinoma-private* (p.R278Q) *FBXW7* alterations. Sample AC15 had a *public FBWX7* mutation (p.117delE). Several *KRAS* and *TP53* mutations were also *public* (7/17 for *TP53* and 12/18 for *KRAS*), indicating that these mutations occurred before progression to carcinoma. We also found *PIK3CA* mutations in three samples, two of which were *adenoma-private* (AC29, p.E545K; and AC40, p.E542K). We found no obvious differences in mutational patterns among carcinoma stages (pTis, pT1, and pT2) (Fig. 2a).

### Adenomas can switch into early-stage carcinomas without additional driver mutations

While the transition from adenoma to carcinoma has been proposed to be accompanied by the acquisition of new CRC driver mutations [15], we found this to be true in only 52% of our matched samples, while 48% of the samples showed no additional driver mutations private to the carcinoma.

To validate these results, we investigated whether cross-contamination between adenomas and carcinomas during sampling could have caused a private mutation to appear public. For this, we compared the allele frequencies (AF) of mutations shared between adenoma-carcinoma pairs without *carcinoma-private* alterations. Our analyses show that public mutations are found at similar AF in both matched regions (Additional file 1: Figure S2A and B), which suggests that the lack of *carcinoma-private* mutations in these pairs was not due to cross-contamination. Furthermore, we also explored whether driver mutations were missed in samples without *carcinoma-private* mutations due to the use of a panel with a limited number of genes. For this, we performed whole-exome sequencing (WES) on four samples (AC27, AC31, AC33, and AC34), without finding additional cancer-private *driver gene* mutations (Additional file 1: Figure S2C and WES results in Additional file 4). Our results here show that the lack of carcinoma-private mutations in CRC driver genes does not seem to be due to experimental limitations, such as cross-contamination of samples in close proximity, nor due to limited coverage of the sequencing panel.

Taken together, our results so far indicate that CRC tumors may not necessarily accumulate additional driver mutations during the switch from adenoma to carcinoma. However, in those cases where carcinoma-private mutations were found, *TP53* mutations were most often involved in the progression from adenoma to carcinoma, followed by *KRAS* and *FBXW7.*

### Positive selection found in both adenoma and early carcinoma

As no additional drivers were found in the four samples investigated using WES, we asked whether we could detect any selective advantage in these paired samples by investigating the burden of non-synonymous and synonymous mutations [13]. We found that in the WES datasets, non-synonymous mutations were more abundant than synonymous mutations in both adenomas and carcinomas (Additional file 1: Figure S3A), hinting towards the potential occurrence of positive selection in both types of neoplasms. To broaden these analyses, we estimated the ratio of the non-synonymous mutations per non-synonymous site to the number of synonymous mutations per synonymous site (dN/dS) in 12 panel-sequenced samples (only samples with matched healthy control tissue were used here) by grouping all 12 adenomas together and comparing them to their matched 12 carcinomas. Our results showed similar dN/dS values of 2.5 for both adenomas and carcinomas, suggesting the action of positive selection in both types of neoplasms (Additional file 1: Figure S3B).

### TP53 gene and protein are already altered in high-grade adenoma

As *TP53* was found to be the earliest driver gene mutated in the switch from adenoma to carcinoma, we next investigated its mutational pattern in greater detail. Public *TP53* mutations were seen in seven samples (Fig. 2a). A majority of these cases, with the exception of AC10 with a CDX2 mutation, did not have any additional driver mutations in the matched carcinoma. Interestingly, when grouped according to adenoma grade, we found that six out of seven samples with *public TP53* mutations were high-grade dysplasia, as shown in Fig. 2b, where only alterations found in the adenoma are displayed. In AC1, a *TP53* alteration present in the adenoma (p.C141Y) was not found in the carcinoma; rather, the latter contained another cancer-private *TP53* alteration (p.N310Tfs*23). Therefore, we found public *TP53* mutations occur more often in high-grade than in low-grade adenomas (*p* = 0.0373 Fisher’s exact).

**Fig. 3:**
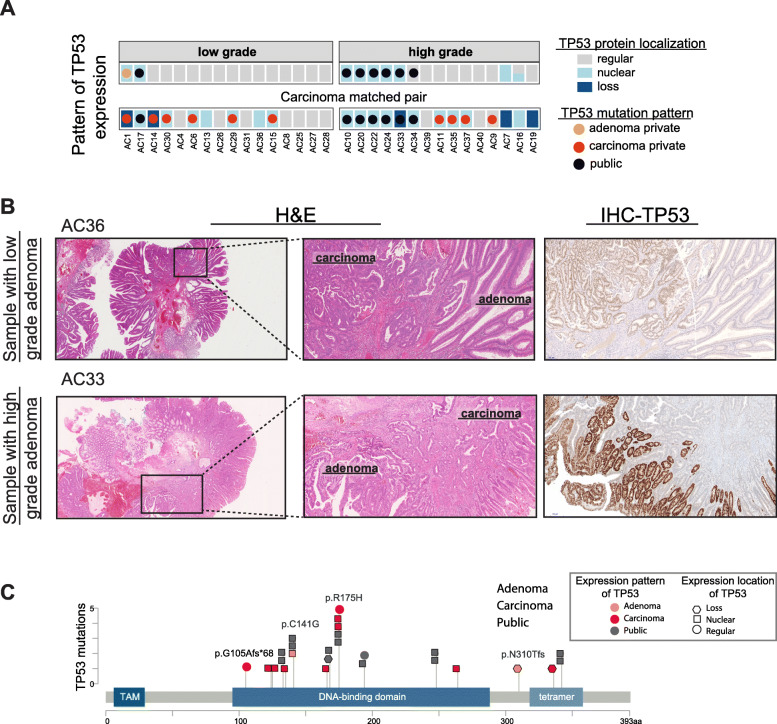
TP53 mutation and expression are altered in high-grade adenoma. **a** Overview of TP53 expression patterns with mutation information from MSS samples. Gray represents regular expression, light blue corresponds to nuclear accumulation, and dark blue is a loss of expression. See the “Methods” section for grading of TP53 staining. **b** Representative H&E and immunohistochemistry (IHC) images of TP53 expression in samples with low-grade (AC36) and high-grade (AC33) adenomas. H&E squares in the left-hand panels are magnified in the middle panel. **c** Location of mutations within the *TP53* gene along with the expression/staining pattern. TAM, transactivation motif (figure is made using MutationMapper Cbioportal)

While the wild-type TP53 protein is an unstable protein with a short half-life displaying immunohistochemistry (IHC)-based scattered or normal detection, the mutant TP53 protein can bind to its wild-type form, stabilizing it in the tumor cells. This leads to its nuclear accumulation and therefore becomes easily detected by IHC [16]. Complete loss of TP53 expression and, therefore, function (often the case in CRC) can also be detected by loss of IHC staining. To assess the changes in TP53 activity during the transition to CRC, we used IHC to visualize protein localization on tissue sections from all samples and compared the changes in protein expression to mutational patterns (Fig. 3 and Additional file 2). We found that *TP53* mutations were frequently accompanied by a strong nuclear staining pattern in the adenomas, including in two low-grade (AC1 and AC17) and five out of six high-grade adenomas with public *TP53* mutations. We also found two high-grade adenomas with no *TP53* mutation but with a TP53 nuclear staining pattern (AC7 and AC16), and a *TP53* public mutation with a regular IHC pattern in sample AC34. TP53 staining was lost in five carcinoma samples. The mutation patterns of *TP53* corresponded to changes in protein accumulation or localization, which were more common in high-grade adenomas (7/15 samples) and carcinomas (22/31 samples) compared to low-grade adenomas (2/16 samples). Representative cases are displayed in Fig. 3b, where a low-grade adenoma (AC36) shows regular TP53 staining, with the adjacent carcinoma tissue exhibiting increased expression and nuclear accumulation. In high-grade adenomas, we found nuclear accumulation of TP53 (AC33) and complete loss of expression in the adjacent carcinoma. Additionally, we show that the localization of TP53 mutations is independent of tissue type and staining pattern (Fig. 3c). These results suggest that *TP53* mutations together with altered protein localization appear in high-grade adenoma. Therefore, TP53 deregulation at multiple levels occurs earlier than the adenoma-to-carcinoma transition. Thus, although the transition between adenoma and carcinoma is regarded as the key first step in CRC progression, our data supports a refined model that emphasizes a role for differences between low- and high-grade adenomas, with the latter sharing key genetic traits with carcinomas, such as *TP53* mutation accompanied with altered expression.

### Chromosome 20 amplifications are found in high-grade adenomas and early carcinomas

**Fig. 4:**
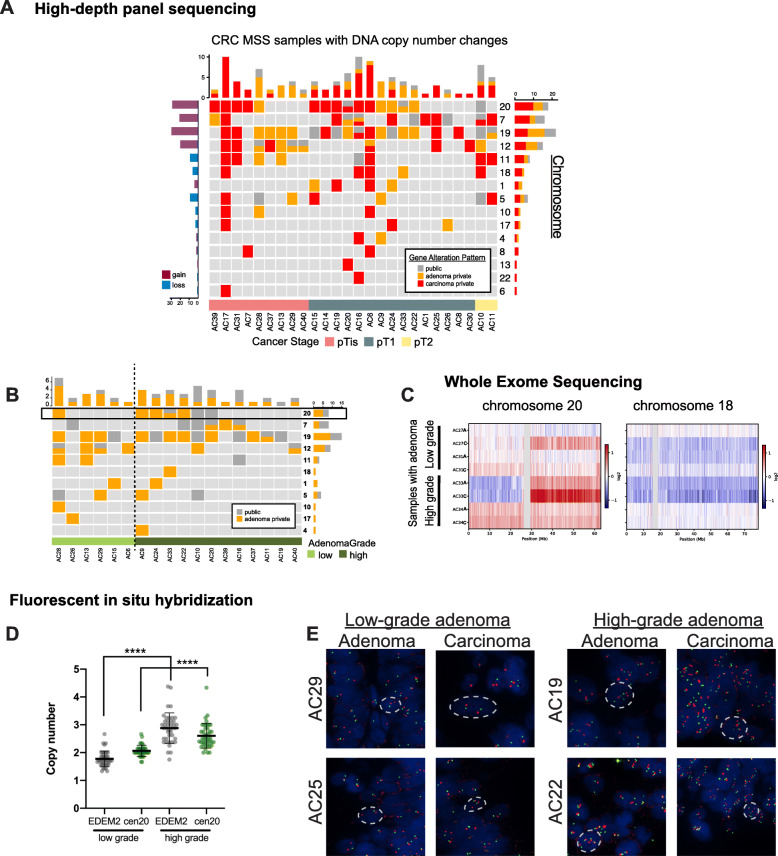
Copy number alterations (CNA) in chromosome 20 are compounded in high-grade adenoma. **a** CNA was analyzed using high-depth panel sequencing data. CNAs in paired samples were labeled public if the same copy number change (either loss or gain) was found in both the adenoma and the carcinoma isolated from the same sample (gray), or only in adenoma or carcinoma from one sample (orange and red, respectively). Gain and loss in DNA copy number on each chromosome across all samples are depicted in purple and blue, respectively. CNAs from samples grouped by carcinoma stage and **b** adenoma grade at the time of isolation. Note, samples without CNA data from panel sequencing are not shown (namely, AC4, 27, 34, 35, 36). **c** A closer look at chromosomes 20 and 18 using WES data reveals the extent of amplification. Samples AC27 and AC31 were low-grade adenomas while AC33 and AC34 were high-grade adenomas. **d** Quantification of FISH from 16 samples using, where possible, up to 40 cells randomly selected within neoplasia-marked regions. Copy number of *EDEM2* gene and centromere 20 (cen20) mean and standard deviation are shown. Unpaired test with Welch’s correction ****p < 0.0001. **e** Representative images from fluorescent in situ hybridization (FISH) analyses for EDEM2 (orange) and chromosome 20 centromere (green) on 4 samples with either low- or high-grade adenoma

Upon investigation of copy number alterations (CNAs), we found the most frequent changes in the MSS samples occurred on chromosomes 19, 20, 12, and 7 with chromosome 20 exhibiting a CNA that was most frequently private to carcinoma (Fig. 4a; Additional file 1: Figure S4A for gene information). The results for MSI samples are shown in Additional file 1: Figure S1C. We also looked at different carcinoma stages to identify the onset of CNAs during CRC progression and found that chromosome 20 amplifications were present in all the carcinoma stages analyzed, including pTis (Fig. 4a). This result indicates that a gain in chromosome 20 is found in the earliest stages of carcinoma development.

We grouped samples which displayed CNAs by grade of adenoma dysplasia and found chromosome 20 amplifications more frequently in high-grade adenomas compared to low-grade adenomas (Fig. 4b). As CNA information from panel sequencing is limited, we additionally used WES, Chip Array, and fluorescent in situ hybridization (FISH) to further analyze the observed changes in chromosome 20. WES from four samples showed that high-grade adenomas (AC33 and AC34) exhibited chromosome 20 amplifications that were not found in low-grade adenomas (AC27 and AC31). Additionally, amplifications of chromosome 20 predominantly involved its long arm. In contrast, loss of chromosome 18q, a well-established cytogenetic aberration in CRC, was not correlated to adenoma grade (Fig. 4c). We also analyzed CNAs using a bead array (MethylationEPIC BeadChip) assay in four samples offering a much higher resolution and a genome-wide view of CNAs [17, 18]. High-grade adenoma samples displayed a clear amplification of chromosome 20, which also extended to the carcinoma samples. In contrast, the low-grade adenomas displayed no changes in chromosome 20 (Additional file 1: Figure S4B). FISH analyses for chromosome 20 using a centromere probe and a gene probe on the long arm of chromosome 20 (*EDEM2*) also revealed higher chromosome 20 copy number in high-grade adenomas (Fig. 4d, e). Our results thus corroborate the CNA findings from panel sequencing. Taken together, our data assigned progressive gains of chromosome 20 from low- to high-grade adenoma. Our results therefore suggest an important molecular progression step from low- to high-grade adenoma that can involve both gene mutations, such as in TP53, as well as CNAs, such as gains of chromosome 20.

### Multi-regional sampling reveals heterogeneity in CRC driver genes in adenomas

**Fig. 5:**
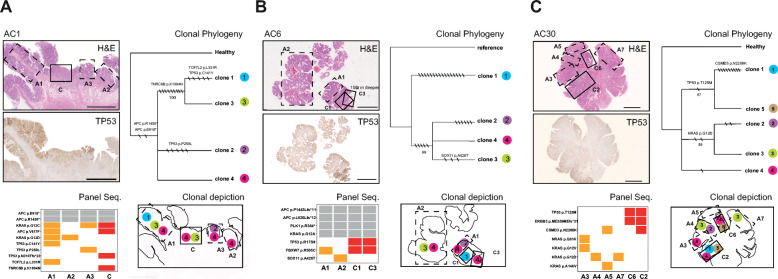
Multi-regional sampling reveals heterogeneity in CRC driver genes in adenomas independent of matched carcinoma. **a**–**c** Three samples were subjected to re-isolations from multiple, histologically defined regions. Each sample has five panels: H&E panel displays a sampling scheme with dotted lines indicating adenoma regions while solid lines represent carcinoma regions separately isolated. TP53 panel depicts the expression of TP53 protein by IHC. Scale bar 5 mm. Panel Seq. results from high-depth CRC panel sequencing where gray indicates public mutations found in all regions, and alterations in adenoma or carcinoma are shown in orange and red, respectively. Clonal Phylogeny panel shows the evolutionary relationship among clones inferred using variants from panel sequencing. The maximum likelihood tree is shown for each CRC sample. Only non-synonymous mutations are labeled; the total number of mutations per branch is indicated by black strikes (synonymous and non-synonymous). Numbers on branches correspond to bootstrap values (1000 iterations) representing nodal support. Note: some mutations are found in panel seq. and not in clonal phylogeny due to analysis discrepancies, whereby for the phylogenetic analyses, we discarded the genomic regions with copy number alterations. Nodes with bootstrap < 50% were collapsed. In the Clonal depiction panel, clonal phylogeny results are overlaid onto an outline of the H&E section to illustrate localization of clones

To further investigate the extent of adenoma heterogeneity found in the matched samples, we sequenced multiple regions within adenomas and their paired carcinomas. To that end, we dissected several histologically distinct areas in three of the MSS samples (Fig. 5; see Table 2 for histological distinctions).

**Table 2:** Histology of samples isolated for multi-regional sampling

Sample	Tissue	Grade (adenoma) Stage (carcinoma)
AC1	Adenoma 1	LG
	Adenoma 2	LG
	Adenoma 3	HG
	Carcinoma	pT1
AC6	Adenoma 1	HG
	Adenoma 2	LG
	Carcinoma 1	pT1
	Carcinoma 3	pTIs
AC30	Adenoma 3	HG
	Adenoma 4	LG
	Adenoma 5	IG
	Adenoma 7	HG
	Carcinoma 2	pTis
	Carcinoma 6	pTis

*LG* low grade, *IG* intermediate grade, *HG* high grade, *pTis* tumor in situ, *pT1* stage 1, *pT2* stage 2

In sample AC1, we isolated three adenoma regions and one carcinoma region (Fig. 5a, H&E panel). Custom panel sequencing identified two public mutations in *APC* (p.E918* and p.R1450*) as well as a private mutation in adenoma region 1 (A1) and cancer region (C) (p.V617F). *KRAS* (p.G12C) is found mutated in all samples except A2, while *KRAS* p.G12D is found in A1 and A2 but not in cancer (Fig. 5a, Panel Seq.). A1 contains an additional *TCF7L2* private mutation. Further, three different *TP53* mutations were also found. A1 harbored p.C141Y, A3 p.P250L, and C p.N310fs*23, while A2 had no *TP53* mutation. In addition, A2 was characterized by loss of TP53 expression (Fig. 5a, TP53 panel).

Sample AC6 was re-dissected into two adenoma regions (A1, A2) of different grades and two carcinomas (C1, C3) of different stages (Fig. 5b), with *public* mutations of *APC*, *KRAS*, and *PLK1* found in all four regions. A2 had an additional *SOX11* mutation, not found in C1 or C3. *FBXW7* mutation was found in the high-grade adenoma region (A1) and in both C1 and C3 carcinoma regions and was similar with regard to CRC driver mutations.

Sample AC30 was dissected into four adenoma regions (A3, A4, A5, and A7) and two pTis carcinoma regions (C2 and C6, with cribriform and tubular histology, respectively) (Fig. 5c). Various RAS mutations with dissimilar AF ranging from 4.5 to 30% were found in the adenomas; however, these mutations were not found in the carcinoma. A3 had *NRAS* (p.Q61K) and *KRAS* (p.G12V) mutations, A4 and A7 carried *KRAS* (p.G12D), and A5 had *KRAS* (p.A146V). All RAS mutations were validated using a smaller commercial panel with an average depth of 1922 reads (Additional file 3) (due to validation using two panels, we allowed a lower cutoff at 4% AF, the clonal phylogeny AF cutoff remained at 8%). Both carcinoma samples had one *TP53* and one *ERBB3* mutation; C2 additionally had a *CSMD3* mutation that was also present in A5. Thus, these multi-regional high-depth panel sequencing results portray heterogeneous adenomas, with several adenoma-specific CRC driver mutations that are not found in the paired, adjacent carcinomas.

### Clonal evolution during adenoma to carcinoma progression

As multi-regional sampling revealed intra-tumor heterogeneity of driver mutations, we next sought to explore the clonal heterogeneity and evolution during CRC progression. We first attempted an evolutionary analysis using panel and WES datasets. As a result, we detected a maximum of three clones (Additional file 1: Figure S5), which prevented conclusive evolutionary interpretations.

We then exploited the multi-regional mutational profiles obtained for samples AC1, AC6, and AC30 (Fig. 5). Here, the clonal deconvolution algorithm revealed four clones in sample AC1, with clone 4 presenting all four regions sampled (A1, A2, A3, and C) (Fig. 5a, Clonal Phylogeny panel). Interestingly, we found two clones (3 and 4) in the carcinoma that did not share a most recent common ancestor; rather, clone 3 was also present in A1 and was most closely related to clone 1, which was only found in the adjacent adenoma region A1. Similarly, in sample AC6, we observed that clones present in the carcinoma did not form a monophyletic group but instead contained a clone (clone 1) not found in either adenomas or matched carcinoma C3 (Fig. 5b, Clonal Phylogeny and Clonal depiction panels). Note such clonal relationship could not be observed using panel sequencing results; for example, in sample AC6, the panel mutations (Fig. 5b, Panel Seq. panel) indicate no differences were found between C1 and C3, unlike the results from clonal phylogeny investigation. In sample AC30, we identified five clones, of which clones 1 and 5 found only in cancer samples (C2 and C6) formed a clade. Here, clone 4 was present in both adenoma and carcinoma regions (A3, C2, C6) (Fig. 5c, Clonal Phylogeny and depiction panels), linking A3 to the rest of the sample, which was not obvious from panel sequencing results alone. Taken together, the results suggest carcinomas do not necessarily originate from a single clone in the adenoma. Additionally, the clones found in the adenomas appear to be heterogeneous, with driver mutations not found in the matched carcinomas.

Furthermore, these results underscore the power of multiple sampling for the study of adenoma/carcinoma evolution, where despite a limited number of mutations, the multi-regional analyses allowed us to detect evolutionary patterns that were not obvious in the analyses of panel datasets for single samples.

## Discussion

In this study, we analyzed DNA mutations and copy number alterations in sets of matched adenoma-to-carcinoma samples. We show that *TP53* mutations and chromosome 20 copy gains distinguish matched adenoma and carcinoma pairs, but that the same mutations are also frequently found in high-grade adenomas. Our study shows that high-grade adenomas possess a molecular genotype that is strikingly similar to that of early-stage carcinoma. In addition, our work suggests that selective pressure might act on both, adenoma and carcinoma, and that the adenomas are usually multi-clonal with particular clones shared between adenomas and carcinomas. Importantly, our analyses show that in the transition of adenoma to CRC, the molecular progression is frequently uncoupled from histological progression. Our data also draws a line between low-grade adenomas, which likely harbor sets of mutations that are insufficient for complete malignant transformation, and high-grade adenomas that already contain a full set of oncogenic drivers allowing transformation to carcinoma.

Among the cancer-specific mutational events commonly encountered in our samples, those predominantly in *TP53*, and to a lesser extent in *FBWX7*, could discriminate between carcinoma and its precursors. Interestingly, a majority of adenomatous precursors with *TP53* mutations displayed high-grade dysplasia, indicating that *TP53* mutations may precede the morphologically visible switch from adenoma to carcinoma. This hypothesis is in agreement with a previous study that showed an association between TP53 expression in adenomas and concurrent carcinomas [19], and with the findings of Hao et al., that describe *TP53* mutations in colorectal adenomas to be dependent on the severity of dysplasia [20]. We additionally find chromosome 20q gains in high-grade adenomas. Gains in chromosome 20q copy numbers are an indicator of poor prognosis in colorectal cancer [21] and are associated with an increased chance of liver metastases [22]. Our samples show both early *TP53* mutations and chromosome 20q gains can already be present in some adenoma and therefore retain the ability to switch to carcinoma without additional driver mutations, similar to the “born-to-be-bad” model suggested by Sottoriva et al. [23].

Our study highlights the importance of using paired adenoma-carcinoma samples to understand CRC progression. Previous studies have investigated large cohorts of unmatched carcinomas and adenomas [20, 24], or adenoma and carcinomas from the same patient isolated from different locations in the colon [25]. While Hao et al. concluded that *TP53* mutations in adenomas are generally different from those seen in carcinomas [20], we find that mutations in codons 248 and 175 are instead often shared among them (Hao et al. identified them to be quite rare in adenomas (1 adenoma and 108 carcinomas with codon 248; 6 adenomas and 83 carcinomas with codon 175)). Importantly, the fate of adenomas must be taken into account; a study for more than 3 years by Pickhardt and colleagues [26] revealed that adenomas can undergo apparent resolution (10%), remain stable (50%), or continue to develop into carcinoma (22%). Thus, the cohorts chosen by Hao et al. and other studies that used unpaired samples perhaps indirectly selected for adenomas that are unable to progress to carcinoma. A recent study by Druliner et al. highlights such genetic differences between adenomas with and without malignancy [27].

Other paired adenoma-carcinoma studies exist, albeit with a limited number of samples. For example, Kim et al. investigated four cancer-in-adenoma samples using WES and concluded that the sequence of mutational events can be different in each sample; however, they investigated only high-grade dysplasia and late-stage carcinoma samples [28]. Nonetheless, similar to our results, they found similar mutational profiles in paired adenoma and carcinoma samples. On the other hand, two studies with paired adenoma and carcinoma samples but with analyses of only *TP53* [29] or *APC*, *KRAS*, and *TP53* [30] concluded that *TP53* was not singularly responsible for switching an adenoma to carcinoma. Difficulties in dissecting adenomatous from cancerous tissue can hamper a clear separation of both components, but comparative analyses of paired adenomatous precursor tissue and its subsequent carcinomatous descendants have been successfully performed in previous studies (as discussed above) and in this study. Our findings of significant and recurrent differences between both isolated components clearly show that the adenoma to carcinoma switch of cytological and architectural features of malignancy go hand-in-hand with genetic alterations typically found in more invasive cancers.

The colonic tissue is made up of individual crypt structures that can maintain unique genetic profiles [31]. When we sequenced multiple areas within paired samples, we found intra-tumor mutational heterogeneity in important CRC genes such as *KRAS* and *SOX11*. In some cases, these mutations were absent in the paired carcinoma, indicating the ability of adenomatous tissue to carry driver mutations that are either not inherited by the subsequent carcinoma or arise after carcinoma progression but remain within histologically defined adenomatous tissue. Further whole-genome sequencing of these samples could help to estimate the time of driver acquisition during CRC progression. Our data suggests that within separate crypts, the dysplastic colonic tissue can harbor mutations in driver genes long considered *carcinoma-private*.

Another advantage of using paired adenoma-carcinoma samples is that they facilitate tracing clonal evolution along with CRC progression. Importantly, here, we inferred clonal trees, in contrast to the sample trees described in a previous CRC paired sample study [32]. This distinction is crucial, as tumors are composed of heterogeneous populations, and each sample should be treated as a collection of clones [33]. Our analyses, albeit limited in number, revealed complex patterns of clonal evolution, suggesting that the various adenoma clones can independently become carcinomas, or in other words, that carcinomas may not necessarily be monoclonal in origin. These findings suggest possible alternative treatment strategies should be considered, for example, with handling polyps which may have already taken a genetic, irreversible step in CRC progression.

Notably, the carcinomas investigated here were still embedded within the adenoma and, therefore, represent the earliest step in the adenoma-to-carcinoma transition process; yet, they already contained all common driver mutations (*APC*, *BRAF*, *KRAS*, *TP53*, *SMAD4*, and *PIK3CA* among others) at frequencies comparable to that seen in more advanced carcinomas [5]. Despite these significant observations, a sample size of 31 MSS adenoma-carcinoma samples precludes meaningful statistical evaluation of less frequent mutations, for example, in *LRP1B* and *LRP2*, which are mutated at intermediate frequencies in advanced CRC, but were not found mutated in our samples. This finding poses the question whether progression to advanced cancer is a matter of continuous growth, or if additional mutations or other layers of genomic deregulation, for example, at the epigenetic level, drive carcinoma development. Our study is in agreement with models of tumor progression that do not rely on the acquisition of further genetic alterations, for instance, on diminishing tumor-suppressive effects by gradual deregulation of the cancer microenvironment [34]. As our study finds evidence of regional and divergent evolution within adenoma, but no clear differences in gene driver mutations between high-grade adenoma and carcinoma, we propose that genetic driver mutations provide a necessary prerequisite for CRC that have to be complemented by further levels of deregulation, such as at the epigenetic level or due to the changes in the micro-environment.

## Conclusions

In conclusion, genetic changes in the colorectal adenoma to carcinoma sequence are uncoupled from histological progression, with high-grade adenomas being able to mimic early-stage carcinomas at the molecular level. These findings emphasize that high-grade adenoma retain genetic alterations required to transform into carcinoma, unlike low-grade adenomas. Whereas large cohorts of colorectal adenomas and unpaired late-stage carcinomas have been repeatedly investigated, a closer look at paired samples in early CRC transformation stages can enhance our understanding of CRC transformation.

## Methods

### Samples and study design

Paired adenoma and carcinoma samples were obtained from the colon and rectum during pathological routine inspection. The samples are termed “matched” as they co-exist in the same polyp or resection sample. All samples displayed histological evidence of neoplastic progression upon microscopic investigation. All patients provided signed consent as part of the clinical documentation protocol of the Charité Universitätsmedizin Berlin. From a total of 34 samples, 22 were excised during polypectomy (endoscopy via colonoscopy) while 12 were obtained by surgical resection owing to polyp position (along the bending colon) or size (too large to remove with polypectomy). We focused on microsatellite stable (MSS) CRC (*n* = 31); however, three microsatellite unstable (MSI) cases were also analyzed for comparison. In several samples, little or no healthy colon tissue could be isolated because routine procedures for sample isolation during polypectomy require minimum disruption of healthy tissue in the colon wall. When present, healthy tissue was used to identify and discard germline single nucleotide polymorphisms (SNPs), otherwise information from the 1000 Genomes Project 1 [35] was used instead. Histologically, 11 MSS samples were premalignant carcinoma in situ (pTis) while 20 submucosa samples were invasive carcinomas (18 pT1 and 2 pT2). Additionally, the paired adenoma component was categorized as either low (*n* = 16) or high (*n* = 15) grade dysplasia.

All samples were formalin-fixed and paraffin-embedded (FFPE). Tissue samples were sectioned and stained with hematoxylin and eosin (H&E). Pathologists demarcated adenoma, carcinoma, and healthy tissue areas in the H&E slides (Additional file 1: Figure S1A), and depending on the size of the marked area in the H&E slides, 5 to 10 sections of 5 μm each were used for DNA isolation. All histological assessments were conducted independently by two pathologists from the institute of pathology at Charité Universitaetsmedizin Berlin.

### DNA isolation

Tissue was macro-dissected from the slides, and DNA was isolated using the GeneRead DNA FFPE kit (Qiagen, Netherlands). The quality and quantity of DNA were determined by TaqMan RNaseP Detection Reagents Kit (Thermo Fisher Scientific, USA). Microsatellite instability was identified using a mononucleotide marker panel (MSI Analysis System, Promega, Germany).

### Targeted massive parallel sequencing

Where possible, 10 ng of DNA was used for panel sequencing with a custom CRC panel (described in Mamlouk et al. [7]). Briefly, the CRC panel covers areas within 100 CRC-related genes, using 793 amplicons covering 125-bp stretches of mainly exon regions. All samples were sequenced using the Ion Torrent PGM technology (Thermo Fisher Scientific, USA). DNA libraries were made by multiplexed PCR amplification with the Ion AmpliSeq Library Kit 2.0 (Thermo Fisher Scientific, USA). Samples were ligated to Ion Xpress Barcode Adapters (Thermo Fisher Scientific, USA) and purified using Agencourt AMPure beads (Beckman-Coulter, USA). Pairs of samples were run on a 316v2 chip, resulting in an average read depth of 1641 (range 539–2955).

For selected results from targeted massive parallel sequencing, we performed Sanger sequencing on specific mutations which we found required validation, for example, due to sub-optimal amplicon performance or location within long nucleotide repeat area. Primer sequences for Sanger sequencing can be found in Additional file 3.

In the case of sample AC30, a smaller commercial panel (Ion Ampliseq Cancer HotSpot Panel V2 (CHP)) (Thermo Fisher Scientific) was additionally used for validation. A final list of mutations comprising non-synonymous single nucleotide variants (SNVs) and small deletions resulting in amino acid changes, loss, or gain of stop codons was assembled (Additional file 3). Fifteen samples displayed no additional private cancer mutations; of these, we chose four samples (AC27, AC31, AC33, and AC34) for whole-exome sequencing (WES) to identify additional private driver mutations.

### Somatic variant calling from targeted massive parallel sequencing

Variant calling was conducted as in [7]. Briefly, we used the PGM variant caller, after which an in-house analysis pipeline, Sofia [36], was utilized to obtain information on nucleotide alterations which may have been called in one sample and not in its paired sample (due to low coverage or quality of DNA). This allows the detailed comparative analysis of paired samples beyond the limitation of investigating only those variants “called” by commercial pipelines. We use a cutoff of 8% allele frequency and quality (*Q*) score of at least 50. In multiple sampling of AC30, we allowed a lower allele frequency cutoff of 4% due to the utilization of two panels for taget sequencing (allowing for extensive depth coverage).

Genetic alterations (somatic genomic alterations, including point mutations, small deletions, and insertions, and copy number aberrations) present in both the carcinoma and its paired adenoma were termed “public,” whereas genetic alterations found exclusively in the adenoma or the carcinoma were termed “private.”

### Copy number analyses from targeted massive parallel sequencing

We utilized panel sequencing results to identify copy number alterations (CNAs) using CNVPanelizer [37] (results in Additional file 5). In short, CNVPanelizer infers CNAs from regions covered by a panel that has been extensively validated previously [7].

### Whole-exome sequencing

Several sample pairs displayed no unique driver mutations in their carcinoma tissue compared with their paired adenoma when using the CRC panel. To find out if our CRC panel missed important driver alterations, we conducted whole-exome sequencing (WES) on four samples; AC33 and AC34 (adenoma, carcinoma, and adjacent healthy) and AC27 and AC31 (adenoma and carcinoma without healthy tissue). DNA from samples (350 ng) was prepared with Low Input Exome-Seq Human v6 library preparation kit (Illumina, USA) according to the manufacturer’s protocol. We performed 125-bp paired-end sequencing on the Illumina HiSeq 2000 v4 according to the manufacturer’s protocol. One library was prepared for each sample and distributed equally onto 3 lanes, with each lane carrying all the samples. The average sequencing depth was 70× (range 62–80×) for tumor and normal samples (results in Additional file 4).

### Somatic variant calling from whole-exome sequencing

Each of the sample was aligned to the reference human genome GRCh37, and the sample BAM files from each lane were merged to generate the starting BAM files of a sample. Further pre-processing of the raw data was done according to best practice by the Genome Analysis Toolkit recommendation. Briefly, the merged BAM files were sorted and marked for read duplicates and then further underwent base realignment and base score recalibration. The newly processed BAM file was then indexed and utilized for the downstream variant analysis.

#### Private mutation calling

Somatic mutation calling of each adenoma tissue to its matched cancerous tissue and vice versa was performed using GATK’s Mutect2 software with an alteration to the default settings. Specifically, the threshold of “max_alt_alleles_in_normal_count” parameter was set to allow the presence of the alteration at 10% in the normal tissue, accommodating for tumor contamination in the normal tissue and contamination of the adenoma-carcinoma pairs in each other. Elimination of most of the false positive was performed in the downstream analysis:

1. Segmental duplication regions were discarded from further analysis.
2. One thousand genome SNPs from the European population with a frequency > 5% were removed.
3. Variant’s allele frequency must be 2 times higher in tumor tissue compared to the control/comparing tissue.
4. There must be at least 3 reads supporting the tumor variant.
5. Allelic frequency of the tissue under consideration must be greater than 4%.
6. FFPE artifacts were also removed utilizing a custom pipeline provided by the DKFZ facility (https://github.com/DKFZ-ODCF/DKFZBiasFilter). In general, the pipeline determines the variants that are generated due to the bias or overrepresentation of the variant on one strand. It also flags for PCR bias, further eliminating false positives.
7. Finally, variants with a filter “PASS,” “homologous mapping event,” and “clustered events” were kept for annotation.

A variant passing all the filtering parameters in the adenoma (non-cancerous) tissue when compared to the carcinoma (cancerous) tissue (variant calling: Mutect2 with the control tissue as carcinoma) of the same patient is designated as “adenoma-private” variant. A variant passing all the filtering parameters in the carcinoma (cancerous) tissue (variant calling: Mutect2 with the control tissue as adenoma) when compared to the adenoma (non-cancerous) tissue of the same patient is designated as “carcinoma-private” variant.

#### Public mutation calling

For samples with normal tissue (AC33 and AC34), somatic mutation calling of adenoma and carcinoma when compared to normal was performed as described above. For samples without normal tissue (AC31 and AC 27), we used VarScan’s single-sample variant calling tools. Pileup2snp and pileup2indel were used to determine any changes in the tumor when compared to the reference genome. The default setting was used for the initial variant calling, and further downstream analysis was performed as follows:

1. Segmental duplication regions were discarded from further analysis.
2. One thousand genome SNPs from the European population with a frequency > 5% were removed.
3. Allelic frequency of the tissue under consideration must be greater than 40%, potentially eliminating many of the patient-specific SNPs.
4. Any SNP called due to a potential indel nearby was also removed.
5. Variants passing these filters were considered tumor-specific variants and were kept for annotation.

Variants which pass all filtering parameters and are present in both adenoma and carcinoma tissue within the same patient were designated as “public” variants.

All the resulting variants were annotated with ANNOVAR software utilizing the human genome GRCh37. Non-synonymous alterations included frameshift insertion and deletion, stop gain, and only insertions and deletions.

### Copy number analysis from whole-exome sequencing

In order to determine segmental copy number variations from whole-exome data, CNVkit [38] software was utilized using the two normal controls, pooled together, as reference. The tool was used in the default setting with the default segmentation algorithm circular binary segmentation (CBS). Bins with low coverage were dropped from the analysis to reduce potential false-positive predictions. Results can be found in Additional file 5.

### Immunohistochemistry

All MSS samples were subjected to immunohistochemistry (IHC) for TP53 expression. Briefly, slides were de-paraffinized using xylol, and antigen retrieval was conducted using citrate buffer. Monoclonal mouse antibody TP53 (Dasko cat#M7001, Agilent, USA) was used at a concentration of 1:50 with overnight incubation at 4 °C, followed by secondary antibody (Dako cat# K500111-2) incubation at room temperature for 1 h. Due to the heterogeneity of TP53 expression, we used a “majority pattern” to describe the expression, as judged by pathologists from our institute. Regular expression was defined as “when not all tumor cells were stained but when those that stained showed heterogeneous intensity, resembling the expression pattern in non-dysplastic colon.” Mutation-typical patterns were scored when either all tumor cells were devoid of TP53 staining (loss) or when TP53 was strongly present in at least 90% of tumor cell nuclei (nuclear).

### Fluorescence in situ hybridization

Fluorescence in situ hybridization (FISH) was performed on 3-μm tumor sections from 10 samples. We used commercially available, standardized, probes for detecting *EDEM2* (Empire Genomics, USA), which is located on the long arm of chromosome 20. Hybridization was performed according to the manufacturer’s instructions. Where possible, we scored 30 cells per sample using an Olympus microscope. The analysis was conducted using the “BioView System, Solo Software” (Abbott Molecular). *T* test with Welch corrections was used for statistical analysis.

### Copy number analyses from EPIC methylation bead chip

One hundred nanograms of FFPE DNA was used with the Infinium HD FFPE DNA Restore Kit (Illumina, San Diego, USA) and analyses performed using the Illumina Infinium MethylationEPIC BeadChip (Illumina, San Diego, USA) according to the manufacturer’s protocols. We calculated the copy number profiles from Illumina EPIC arrays using the “conumee” package for R (http://bioconductor.org/packages/conumee).

### Non-synonymous/synonymous alterations analysis

In order to estimate the ratio of the number of non-synonymous mutations per non-synonymous site to the number of synonymous mutations per synonymous site (dN/dS), we used the dNdScv R package by Martincorena et al. [39], a maximum-likelihood dN/dS method designed to quantify selection in cancer and somatic evolution. This tool was designed to work with cohorts of patients in order to have enough number of mutations. Thus, we created one adenoma cohort and one carcinoma cohort merging together all the samples sequenced by CRC panel with matched normal. We removed those variants found in the matched normal sample to keep only the somatic ones. Then, we ran dNdScv passing the genes in the panel to the argument “gene_list.”

### Evolutionary analyses

#### Clonal deconvolution and phylogenetic analyses

After filtering non-diploid regions from panel sequencing variants, we performed clonal deconvolution using LICHeE [40] (details in the “Clonal deconvolution” section). Next, maximum likelihood phylogenetic trees of the clones were constructed using Paup [41], under a Jukes and Cantor model [42] of nucleotide substitution. We assessed the statistical nodal support using bootstrap [43] with 1000 replicates and collapsed nodes with bootstrap < 50%.

#### Clonal deconvolution

For all samples analyzed, we first conducted clonal deconvolution using LICHeE [44]. Three samples from the original panel sequencing cohort (AC13, AC14, and AC35) were used together with their healthy control samples. Two samples (AC33 and AC34) from the samples analyzed by whole-exome sequencing were used because they had a healthy control while the others did not. Three samples from multiple-sampling panel sequencing (AC1 and AC30 with healthy control tissue, and AC6 with reference human genome as healthy control) were used. To select variants for the evolutionary study, we followed the steps summarized below.

##### Selecting variants from panel sequencing

We used the variants called by the PGM caller. We selected the PASS SNVs (removed indels and MNPs) in diploid regions (using the copy number data provided by CNVkit (see above)). Then, we recovered the read counts in those positions for all the samples (including the healthy one if available) using GATK CollectAllelicCounts. We selected variants reliable for clonal deconvolution following these criteria:

- Minimal depth of 20× in all samples. It is possible for a variant to be present in one sample but not be called due to the lack of sequencing depth.
- Minimal VAF of 0.04 in at least one sample. This threshold was selected according to the limit of detection of the panel.
- For patients without a healthy sample, variants present in dbSNP are considered germline and thus discarded.
- For those patients with a healthy sample, variants with VAF above 0.1 in the healthy sample are considered germline and thus discarded.
- For AC6 we included chr7:2976771.
- In the case of multi-allelic variants, if both alternative alleles show similar frequencies (the ratio of the read counts for minor_alt_allele/major_alt_allele is higher than 0.2), the variant is discarded. Otherwise, only the major alternative allele is kept.

We ran LICHeE with the following parameters:

**Table:** 

Parameter	Value
-minVAFPresent	0.1
-maxVAFAbsent	0.04
-maxVAFValid	0.6
-minClusterSize	1
-maxClusterDist	0.2
-e	0.1

Next, we parsed the outputs from LICHeE to get the sequences of the clones, using an in-house script.

##### Selecting variants from whole-exome sequencing

We used the variants called against that healthy by Mutect2 in the adenoma and/or the carcinoma. Thus, germline variants were excluded during the somatic variant calling process. We selected the PASS SNVs (removed indels and MNPs) in diploid regions (using the copy number data provided by CNVkit (see above)). Then, we recovered the read counts in those positions for both adenoma and carcinoma using GATK CollectAllelicCounts. We selected variants reliable for clonal deconvolution following these criteria:

- Minimal depth of 20× in all of the samples. It is possible for a variant to be present in one sample but not be called due to the lack of sequencing depth.
- Minimal VAF of 0.05 in at least one sample.
- In the case of multi-allelic variants, if both alternative alleles show similar frequencies (the ratio of the read counts for minor_alt_allele/major_alt_allele is higher than 0.2), the variant is discarded. Otherwise, only the major alternative allele is kept.
- For AC34 we also removed the variant in 12:50745779 since it looks like a germline SNP not detected by Mutect2. The allele frequency is 0.45 in adenoma and 0.4 in carcinoma, and the tumor purity of the sample is low. This alteration is reported as a synonymous SNP in dbSNP (rs766535208).

After the filtering, we obtained 110 variants for AC33 and 244 variants for AC34. We ran LICHeE with the following parameters:

**Table:** 

Parameter	Value
-minVAFPresent	0.05
-maxVAFAbsent	0.025
-maxVAFValid	0.6
-maxClusterDist	0.15
-e	0.1
-minClusterSize	1

Next, we parsed the outputs from LICHeE to get the sequences of the clones, using an in-house script.

All the scripts for the evolutionary analyses are available at https://github.com/lauratl/AdeCar.

## Supplementary information

**Additional file 1: Supplementary figure S1-S5.** **FigS1.** Adenoma/carcinoma samples and MSI analysis. **FigS2.** Allele frequencies of mutations from panel and whole exome sequencing. **FigS3.** Non-synonymous and synonymous mutation analysis. **FigS4.** DNA Copy Number Analysis. **FigS5.** Evolutionary relationship of samples from single-region sampling analysis.

**Additional file 2.** Cohort details.

**Additional file 3.** Panel sequencing mutations.

**Additional file 4.** Whole exome sequencing mutations.

**Additional file 5.** CNA Panel sequencing.

**Additional file 6.** CNA Whole exome sequencing.

## Data Availability

Panel sequence data and BeadChip array data have been deposited at the European Genome-phenome Archive (EGA), which is hosted by the EBI and the CRG, under accession number EGAS00001003468. Somatic mutations from whole-exome sequencing are available in Additional file 4, and VCF files can be made available upon request.

## References

[1] Arnold M, Sierra MS, Laversanne M, Soerjomataram I, Jemal A, Bray F (2017). Global patterns and trends in colorectal cancer incidence and mortality. Gut..

[2] Sievers CK, Grady WM, Halberg RB, Pickhardt PJ (2017). New insights into the earliest stages of colorectal tumorigenesis. Expert Rev Gastroenterol Hepatol.

[3] Vogelstein B, Fearon ER, Hamilton SR, Kern SE, Preisinger AC, Leppert M (1988). Genetic alterations during colorectal-tumor development. N Engl J Med.

[4] Gao J, Aksoy BA, Dogrusoz U, Dresdner G, Gross B, Sumer SO (2013). Integrative analysis of complex cancer genomics and clinical profiles using the {cBioPortal.}. Sci Signal.

[5] Cerami E, Gao J, Dogrusoz U, Gross BE, Sumer SO, Aksoy BA (2012). The cBio Cancer Genomics Portal: an open platform for exploring multidimensional cancer genomics data. Cancer Discov.

[6] Baker SJ, Preisinger AC, Jessup JM, Paraskeva C, Markowitz S, Willson JKV (1990). p53 gene mutations occur in combination with 17p allelic deletions as late events in colorectal tumorigenesis. Cancer Res.

[7] Mamlouk S, Childs LH, Aust D, Heim D, Melching F, Oliveira C (2017). DNA copy number changes define spatial patterns of heterogeneity in colorectal cancer. Nat Commun.

[8] Bläker H, Alwers E, Arnold A, Herpel E, Tagscherer KE, Roth W, et al. The association between mutations in BRAF and colorectal cancer–specific survival depends on microsatellite status and tumor stage. Clin Gastroenterol Hepatol. 2018;17(3):455–462.e6. 10.1016/j.cgh.2018.04.015 29660527

[9] Biswas S, Trobridge P, Romero-Gallo J, Billheimer D, Myeroff LL, Willson JKV, et al. Mutational inactivation of TGFBR2 in microsatellite unstable colon cancer arises from the cooperation of genomic instability and the clonal outgrowth of transforming growth factor β resistant cells. Genes Chromosom Cancer. 2008;47(2):95–106. 10.1002/gcc.20511 17985359

[10] Fearon ER, Vogelstein B (1990). A genetic model for colorectal tumorigenesis. Cell..

[11] Fearon ER (2011). Molecular genetics of colorectal cancer. Annu Rev Pathol.

[12] Chang P-Y, Chen J-S, Chang S-C, Wang M-C, Chang N-C, Wen Y-H (2017). Acquired somatic *TP53* or *PIK3CA* mutations are potential predictors of when polyps evolve into colorectal cancer. Oncotarget..

[13] Martincorena I, Raine KM, Gerstung M, Dawson KJ, Haase K, Van Loo P, Davies H, Stratton MR, Campbell PJ. Universal Patterns of Selection in Cancer and Somatic Tissues. Cell. 2017;171(5):1029–1041.e21. 10.1016/j.cell.2017.09.042 PMC5720395 29056346

[14] Alves JM, Prado-López S, Cameselle-Teijeiro JM, Posada D (2019). Rapid evolution and biogeographic spread in a colorectal cancer. Nat Commun.

[15] Fearon ER and Vogelstein B. A genetic model for colorectal tumorigenesis. Cell. 1990;61:759–67. 10.1016/0092-8674(90)90186-i 2188735

[16] Ando K, Oki E, Saeki H, Yan Z, Tsuda Y, Hidaka G (2015). Discrimination of p53 immunohistochemistry-positive tumors by its staining pattern in gastric cancer. Cancer Med.

[17] Feber A, Guilhamon P, Lechner M, Fenton T, Wilson GA, Thirlwell C, et al. Using high-density DNA methylation arrays to profile copy number alterations. Genome Biol. 2014;15(2):R30. 10.1186/gb-2014-15-2-r30 PMC4054098 24490765

[18] Sturm D, Witt H, Hovestadt V, Khuong-Quang DA, Jones DTW, Konermann C (2012). Hotspot mutations in H3F3A and IDH1 define distinct epigenetic and biological subgroups of glioblastoma. Cancer Cell.

[19] Saleh HA, Aburashed A, Bober P. P53 protein immunohistochemical expression in colonic adenomas with and without associated carcinoma. Am J Gastroenterol. 1998;93(6):980–4. 10.1111/j.1572-0241.1998.00292.x 9647033

[20] Hao XP, Frayling IM, Sgouros JG, Du MQ, Willcocks TC, Talbot IC (2002). The spectrum of p53 mutations in colorectal adenomas differs from that in colorectal carcinomas. Gut..

[21] Mikaberidze A (2007). Gain of chromosome 20q is an indicator of poor prognosis in colorectal cancer. Perspect Terror.

[22] Hidaka S, Yasutake T, Takeshita H, Kondo M, Tsuji T, Nanashima A (2000). Differences in 20q13.2 copy number between colorectal cancers with and without liver metastasis. Clin Cancer Res.

[23] Sottoriva A, Kang H, Ma Z, Graham TA, Salomon MP, Zhao J (2015). A Big Bang model of human colorectal tumor growth. Nat Genet.

[24] Cross W, Kovac M, Mustonen V, Temko D, Davis H, Baker AM (2018). The evolutionary landscape of colorectal tumorigenesis. Nat Ecol Evol.

[25] Vaqué JP, Martínez N, Varela I, Fernández F, Mayorga M, Derdak S (2015). Colorectal adenomas contain multiple somatic mutations that do not coincide with synchronous adenocarcinoma specimens. PLoS One.

[26] Pickhardt PJ, Kim DH, Pooler BD, Hinshaw JL, Barlow D, Jensen D (2013). Assessment of volumetric growth rates of small colorectal polyps with CT colonography: a longitudinal study of natural history. Lancet Oncol.

[27] Druliner BR, Wang P, Bae T, Baheti S, Slettedahl S, Mahoney D (2018). Molecular characterization of colorectal adenomas with and without malignancy reveals distinguishing genome, transcriptome and methylome alterations. Sci Rep.

[28] Kim T-Y (2015). Clonal origins and parallel evolution of regionally synchronous colorectal adenoma and carcinoma. Oncotarget..

[29] Inoue M, Enomoto T, Fujita M, Fukunaga M, Takami K, Yana I (1994). A frequent alteration of p53 gene in carcinoma in adenoma of colon. Cancer Res.

[30] Thirlwell C, Will OCC, Domingo E, Graham T A., McDonald SAC, Oukrif D, et al. Clonality assessment and clonal ordering of individual neoplastic crypts shows polyclonality of colorectal adenomas. Gastroenterology. 2010;138(4):1441–1454.e7. 10.1053/j.gastro.2010.01.033 20102718

[31] Snippert HJ, van der Flier LG, Sato T, van Es JH, van den Born M, Kroon-Veenboer C (2010). Intestinal crypt homeostasis results from neutral competition between symmetrically dividing Lgr5 stem cells. Cell..

[32] Saito T, Niida A, Uchi R, Hirata H, Komatsu H, Sakimura S (2018). A temporal shift of the evolutionary principle shaping intratumor heterogeneity in colorectal cancer. Nat Commun.

[33] Alves JM, Prieto T, Posada D (2017). Multiregional tumor trees are not phylogenies. Trends Cancer.

[34] Bissell MJ, Hines WC (2011). Why don’t we get more cancer? A proposed role of the microenvironment in restraining cancer progression. Nat Med.

[35] Auton A, Abecasis GR, Altshuler DM, Durbin RM, Bentley DR, Chakravarti A, et al. A global reference for human genetic variation. Nature. 2015;526(7571):68–74. 10.1038/nature15393 PMC4750478 26432245

[36] Childs LH, Mamlouk S, Brandt J, Sers C, Leser U (2016). SoFIA: a data integration framework for annotating high-throughput datasets. Bioinformatics..

[37] Oliveira C, Wolf T (2015). Reliable CNV detection in targeted sequencing applications. R package version 1.1.0.

[38] Talevich E, Shain AH, Botton T, Bastian BC (2016). CNVkit: genome-wide copy number detection and visualization from targeted DNA sequencing.

[39] Martincorena I, Raine KM, Gerstung M, Dawson KJ, Haase K, Van Loo P (2017). Universal patterns of selection in cancer and somatic tissues. Cell.

[40] Popic V, Salari R, Hajirasouliha I, Kashef-Haghighi D, West RB, Batzoglou S (2015). Fast and scalable inference of multi-sample cancer lineages. Genome Biol.

[41] Cummings MP. PAUP* (phylogenetic analysis using parsimony (and other methods)). Dictionary of Bioinformatics and Computational Biology. Wiley. 2014.

[42] Jukes TH, Cantor CR (1969). Evolution of protein molecules. Mammalian protein metabolism. Volume III.

[43] Felsenstein J. Confidence limits on phylogenies: an approach using the bootstrap. Evolution (N Y). 1985;39(4):783–791. 10.1111/j.1558-5646.1985.tb00420.x 28561359

[44] Popic V, Salari R, Hajirasouliha I, Kashef-haghighi D, West RB (2015). Fast and scalable inference of multi-sample cancer lineages.

