# Supplementary material for: Malignant transformation and genetic alterations are uncoupled in early colorectal cancer progression
Source: BMC Biol. 2020 Sep 7;18:116. doi: 10.1186/s12915-020-00844-x (PMC7487684; doi:10.1186/s12915-020-00844-x)
Supplement: Supplementary file 1 — Additional file 1: Supplementary figure S1-S5. FigS1. Adenoma/carcinoma samples and MSI analysis. FigS2. Allele frequencies of mutations from panel and whole exome sequencing. FigS3. Non-synonymous and synonymous mutation analysis. FigS4. DNA Copy Number Analysis. FigS5. Evolutionary relationship of samples from single-region sampling analysis. [file 12915_2020_844_MOESM1_ESM.pdf]

Figure S1

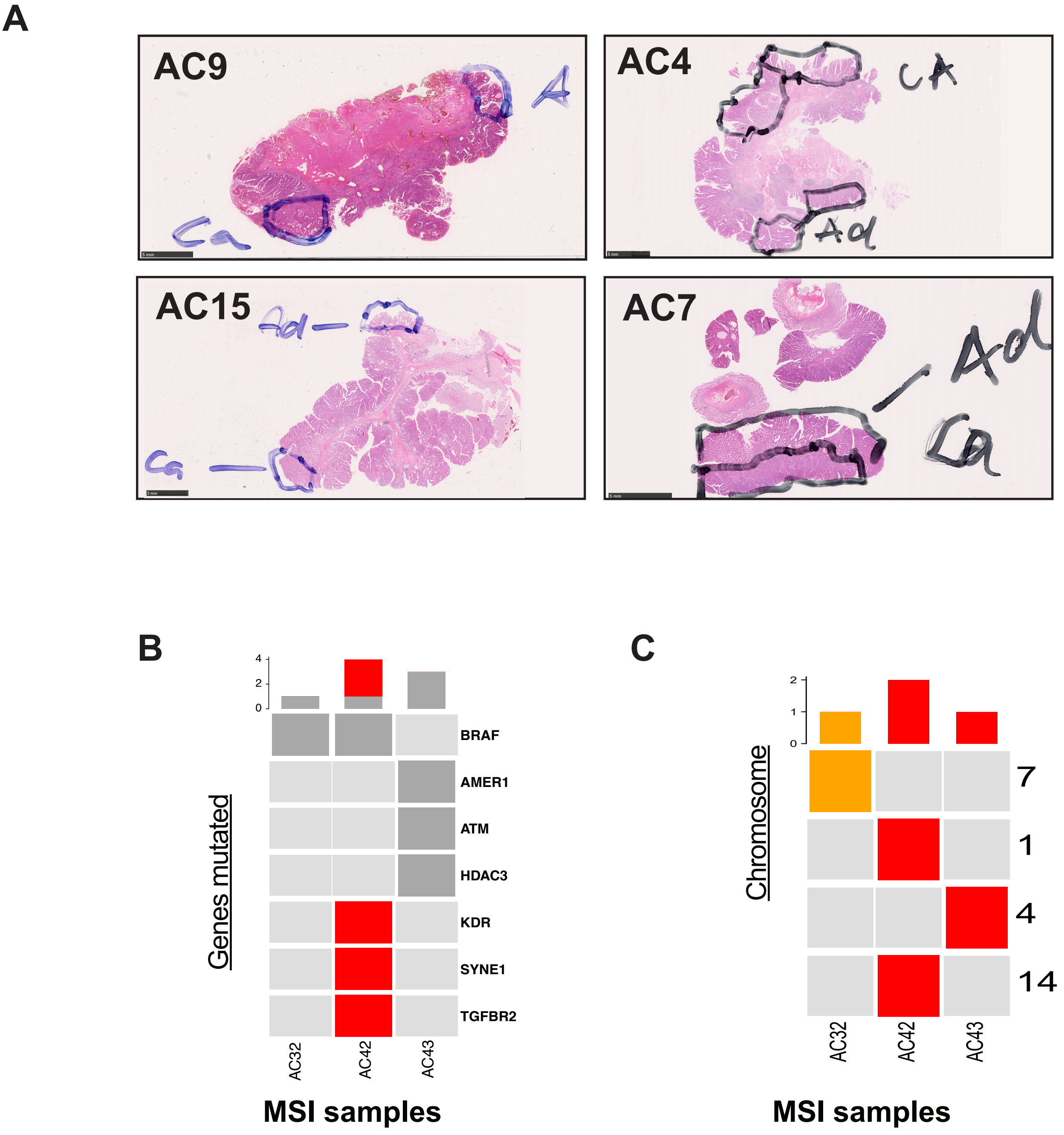

Figure S1. (A) Microscopic view of representative Hematoxylin and Eosin (H&E) sections from adenoma-carcinoma samples used in our study. Markings indicate areas identified by pathologists for DNA isolation. A or Ad marks adenoma tissue, CA carcinoma tissue. Scale bar 5mm. (B) Mutations and (C) Copy number changes in MSI from high depth panel sequencing.

Figure S2

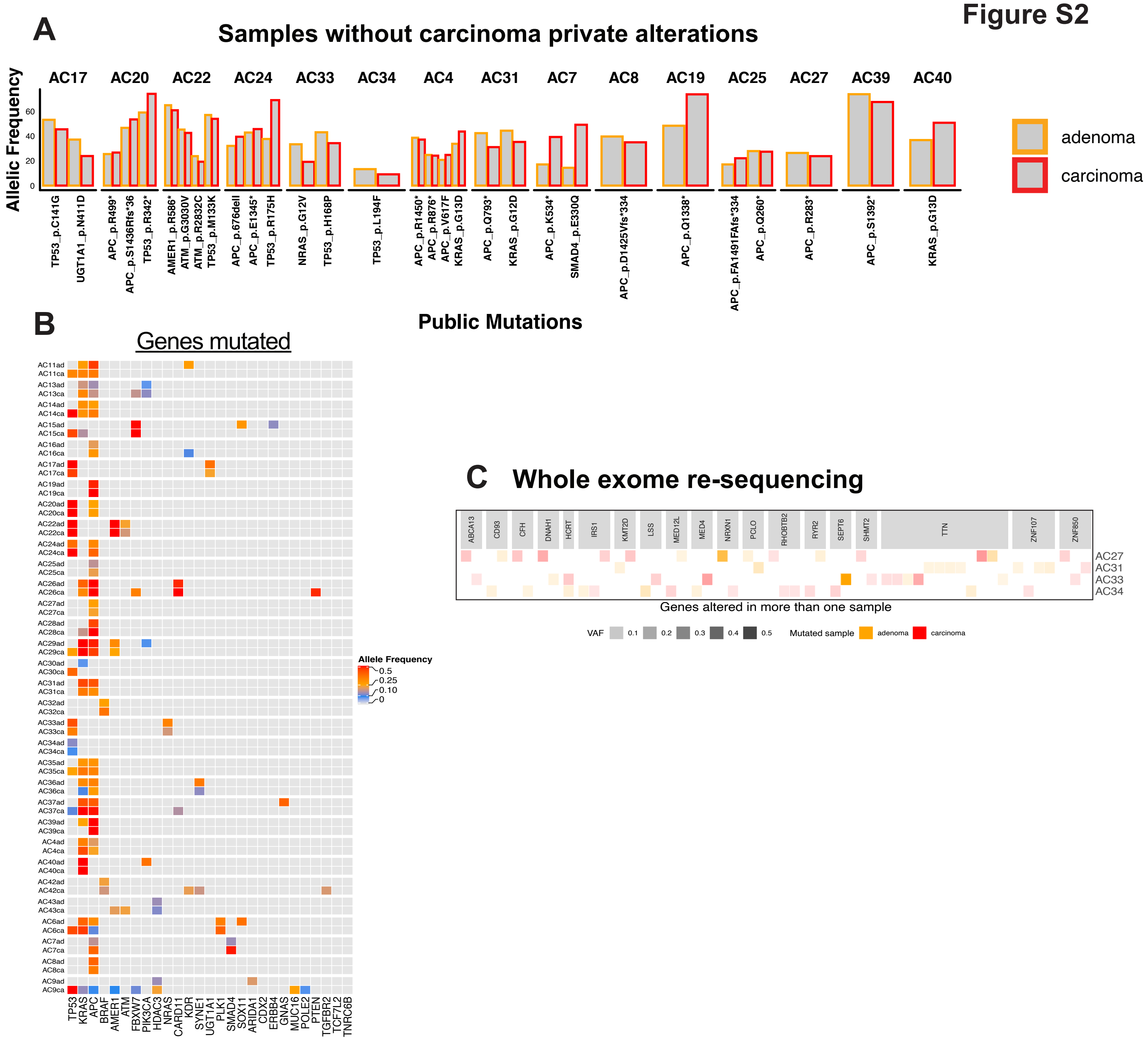

Figure S2. (A) All samples which showed no additional private mutations in the matched carcinoma, are shown here with allele frequencies (AF) of their public mutations. Orange represents AF in the adenoma and red in the carcinoma. AF roughly correlates with amount of mutated tumor cells in a sample. Consequently, contamination of one tissue with mutated cells from neighboring matched tissue would appear as low AF in the first sample and a much higher AF of the same mutation in the matched tissue. In our cohort we found similar AF in the adenoma and carcinoma components of all samples without carcinoma-private alterations. For example, in sample AC17 the AF of TP53 mutation was 53% in the adenoma and 45% in the carcinoma, and for UGT1A1 it was 37% and 24%. Note, in sample AC7 tumor cell content was lower in the adenoma region than in the carcinoma region, as demonstrated by the AF of APC (17% and 39% respectively). (Additional file 3, CRC_Panel_Seq). (B) Graphical display AF of all mutations from CRC panel sequencing. ad is adenoma, ca is carcinoma (C) Four samples re-sequenced by WES, here a representation of mutations found in more than one of the samples. Complete mutation list found in Additional file 4. We found low frequency carcinoma-private alterations in non-driver CRC genes such as KMT2B and RYR2. Additionally, ZNF850 was mutated in 3 samples, however, the low AF (4%, 7.8%, and 4%) compared to higher AF for public mutations (44%, 23%, and 13%) in samples AC31, AC27, and AC33 respectively, implied that it was not a carcinoma driver mutation in the early stage carcinoma samples in our cohort. We find additional public mutations in APC (samples AC33 and AC34). All mutations from WES and AFs are listed in Additional file 4.

Figure S3

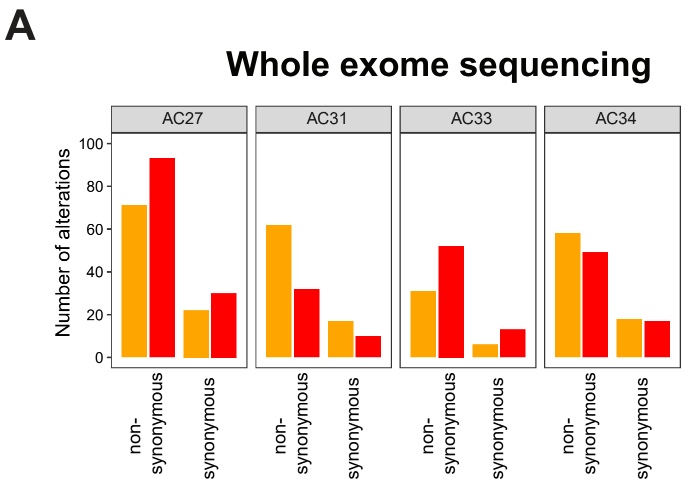

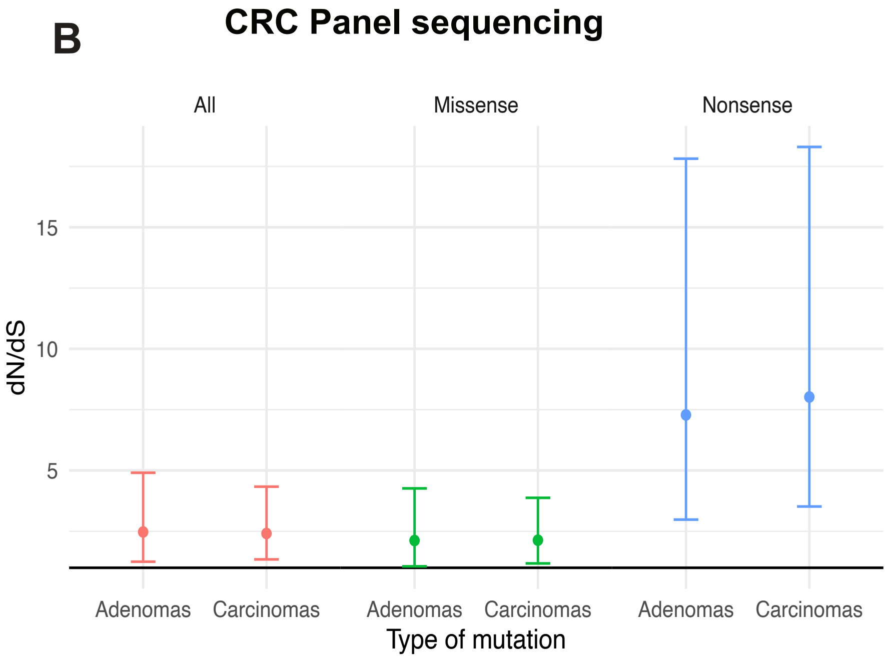

Figure S3. (A) Total number of private non-synonymous and synonymous mutations per sample from WES. Private adenoma mutations are in orange and private carcinoma in red. (B) dN/dS study of all panel sequencing data from 12 samples with adenomas grouped together compared to their matcehd carcinomas grouped together. Only MSS samples with matched healthy tissue are investigated here.

Figure S4

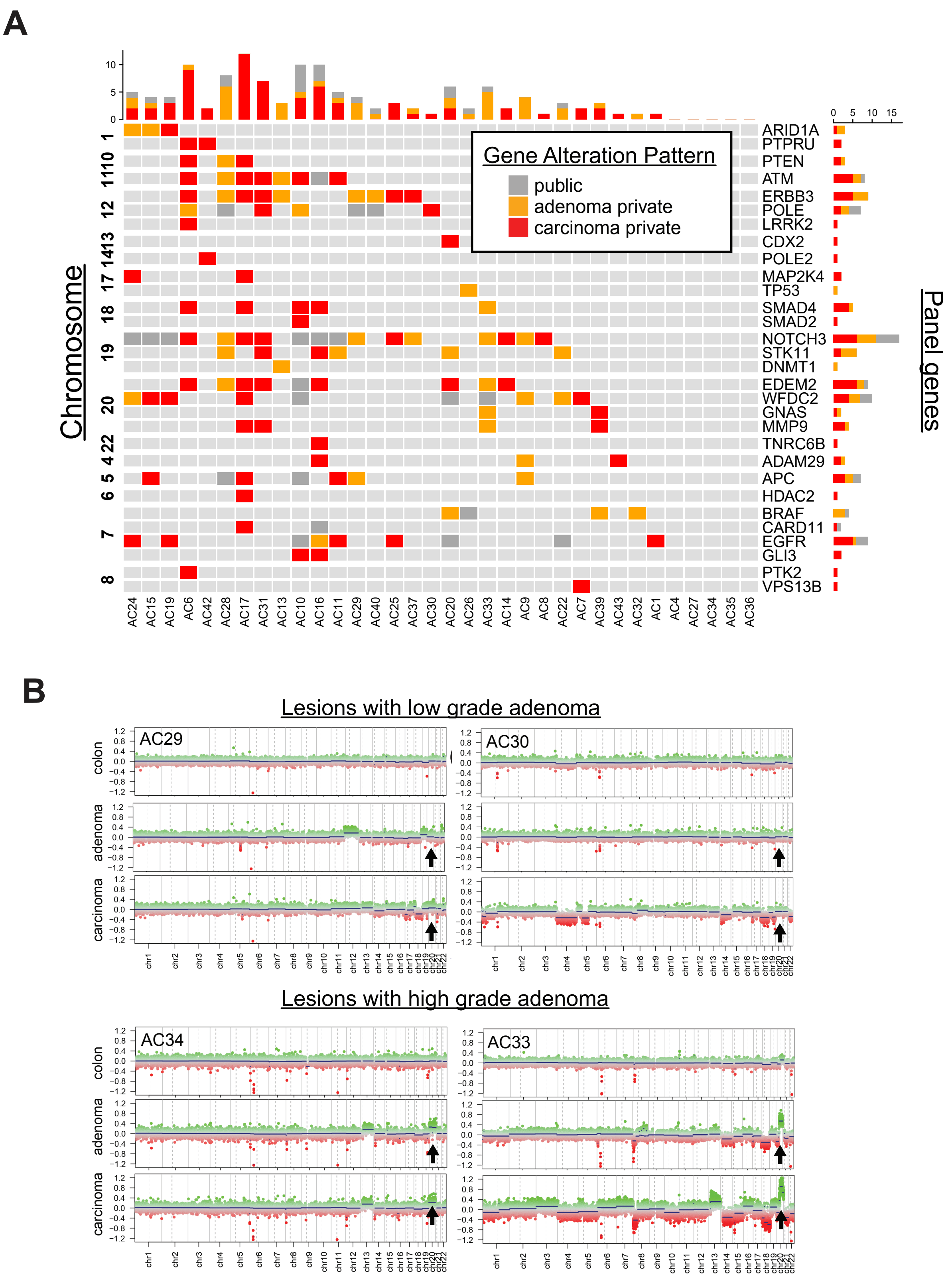

Figure S4. (A) Copy Number Alterations (CNAs) of individual genes from cohort. Note, only MSS samples are shown here. (B) Two AC samples with low grade adenoma (top panels) and two with high grade adenoma (bottom panels) were further investigated at a broader chromosomal level using the 850K EPIC array-based panel. Arrows point at chromosome 20.

Figure S5

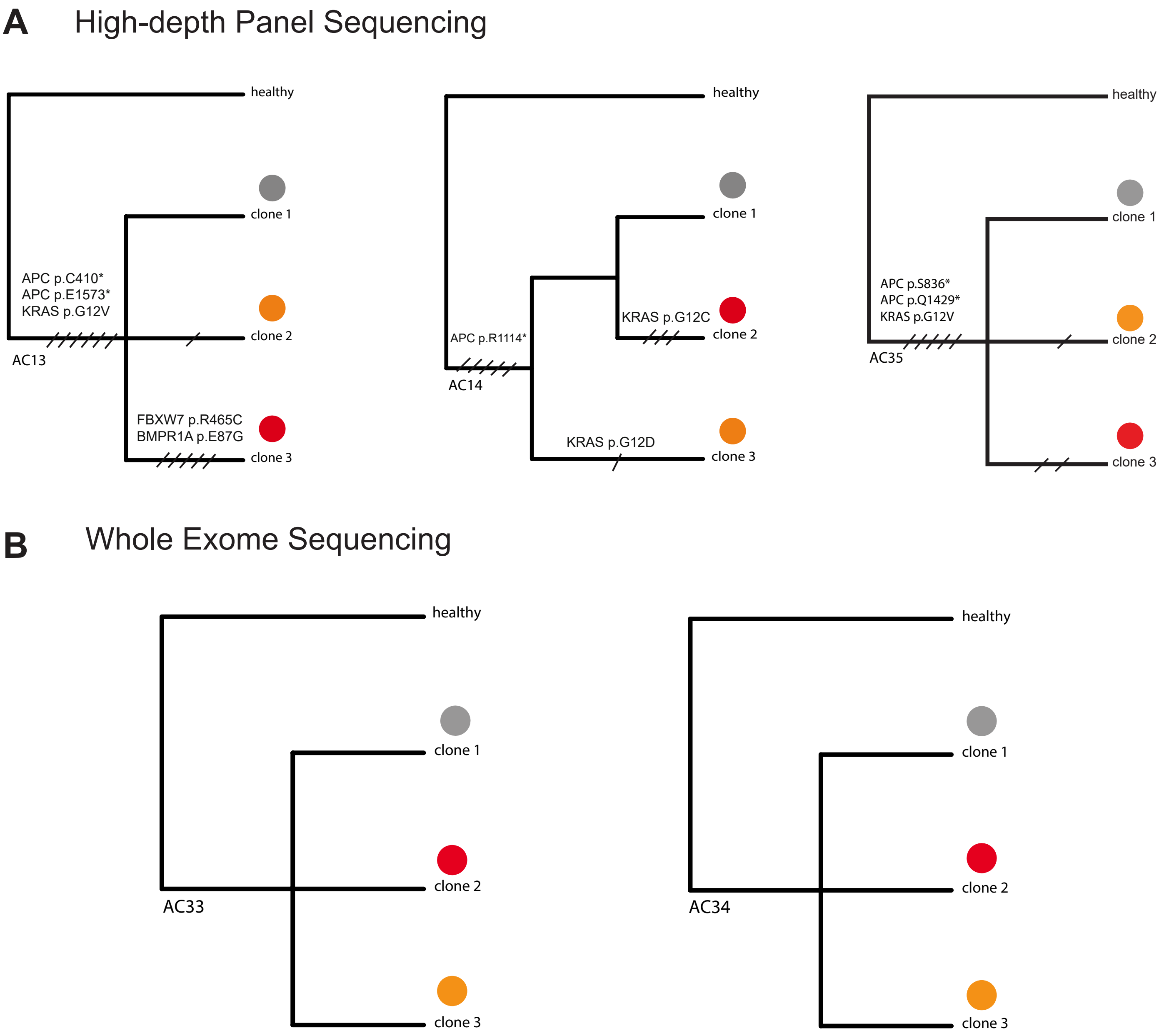

Figure S5 (A) Representative evolutionary relationship of paired adenoma and carcinoma from 3 samples. Variants from panel sequencing. (B) Evolutionary relationship of adenoma and carcinoma from 2 samples. Variants from whole exome sequencing. Only two samples with matched healthy tissue from WES samples utilized. All trees are inferred from maximum likelihood. Colors indicate in which sample the clone was found; grey is public (found in both adenoma and carcinoma), orange and red means found in adenoma only or carcinoma only respectively.
